# Three-dimensional evaluation of root position at the reset appointment without radiographs: a proof-of-concept study

**DOI:** 10.1186/s40510-018-0214-4

**Published:** 2018-06-04

**Authors:** Robert J. Lee, Sarah Pi, Justyn Park, Gerald Nelson, David Hatcher, Snehlata Oberoi

**Affiliations:** 1 Division of Orthodontics, University of California San Francisco, 707 Parnassus Ave. d3000, San Francisco, CA 94143 USA; 2 School of Dentistry, University of California San Francisco, San Francisco, CA USA; 3 Department of Orofacial Sciences, University of California San Francisco, San Francisco, CA USA

## Abstract

**Background:**

Accurate root position is integral for successful orthodontic treatment. Current methods of monitoring root position are either inaccurate, exhibit poor resolution, or use relatively large amount of radiation relative to the benefits for the patient. The purpose of this study was to present an approach that can monitor root position during orthodontic treatment with minimal radiation.

**Methods:**

Cone-beam computed tomography (CBCT) scans were taken for a patient at pre-treatment and at a dedicated reset appointment. An extra-oral laser scan of a poured up cast was taken at the reset appointment. An expected root position (ERP) setup, an approximation of the root position at the reset appointment, was generated using the pre-treatment CBCT scan and reset appointment cast. The ERP setup was compared to the CBCT scan taken at the reset appointment which served as the control. Color displacement maps were generated to measure any differences between the expected and true root positions.

**Results:**

Color map displacement analysis after indirect superimposition found displacement differences of 0.021 mm ± 0.396 mm for the maxillary roots and 0.079 mm ± 0.499 mm for the mandibular roots.

**Conclusions:**

This approach was demonstrated in a patient at the reset appointment to have the potential to accurately monitor root positions during treatment in three dimensions without the need for additional radiographs.

## Background

The goal of orthodontic treatment is to move the teeth into a stable, esthetic, and functional occlusion with every crown and root positioned ideally in three dimensions. To achieve this optimal occlusion, orthodontists often follow Andrews’ six keys to normal occlusion [1]. While four of Andrews’ keys (molar relationship, rotations, spaces, and occlusal plane) are guided by crown position, his other two keys (mesiodistal angulation and buccolingual inclination) depend on both crown and root position. Root position plays a role in the mesiodistal angulation and buccolingual inclination because of variations in crown morphologies, inconsistencies in crown-root angulations, and when a crown is short relative to root length [2–7].

Proper root placement is important for satisfactory periodontal health, restorative treatment, and proper occlusal function. Prior studies have found that if roots of adjacent teeth are placed in close proximity to one another, periodontal or restorative treatment may be compromised [8, 9]. Root proximity and the shape of the crowns are potential causes for a poorly shaped gingival embrasure [10]. Root proximity in which the adjacent roots are 1.0 mm or less apart has been shown to result in jeopardized health of the interproximal space, horizontal bone loss, and more rapid periodontal breakdown [11–15]. Furthermore, other studies have demonstrated that proper root placement and parallelism are critical to distribute occlusal forces and to produce proper occlusal and incisal function [2, 16].

Accurate bracket placement facilitates tooth movement into normal occlusion and minimizes the amount of required wire bending [17]. However, it is difficult to attain dependably accurate placement of all brackets at the initial bonding. To correct for improperly placed brackets that have resulted in improper crown and root positions, the practitioner may either make adjustments in the archwire or reposition the bracket. Carlson and Johnson described an efficient treatment process of using a single dedicated reset appointment to correct any bracket-positioning errors after the initial leveling and aligning of the teeth [18]. At this dedicated reset appointment, both clinical and radiographic examination were performed to assess the position of the crown and roots, and one reason to reposition a bracket would be to address a root parallelism problem. However, an accurate radiographic technique controlling beam angulation or using volumetric imaging is required to assess root parallelism.

Traditionally, panoramic radiographs have been used to monitor and finalize root positions in orthodontic treatment. In a 2008 *Journal of Clinical Orthodontics* (JCO) survey of American orthodontists, 67.4% of respondents reported that they took progress panoramic radiographs and 80.1% of respondents reported that they took post-treatment panoramic radiographs to assess root position [19]. However, panoramic radiographs have been found to be inaccurate in depicting root position through numerous studies which have found that panoramic radiographs have distortions because of the nonorthogonal X-ray beams directed at the teeth [20–23]. Furthermore, prior studies have determined that radiographic techniques should be able to depict root angulations with an accuracy of 2.5° in either direction to be considered clinically acceptable, yet panoramic radiographs depict 53–73% of root angulations outside of this clinically acceptable range [21–24]. A more accurate approach for evaluating root placement, especially at the reset appointment, will facilitate finalizing root positions.

Cone-beam computed tomography (CBCT) is another radiographic technique that is becoming increasingly more common to use in orthodontics. In contrast with panoramic radiographs, CBCT scans have been found to accurately depict true root angulations and inclinations in three dimensions and show dentofacial structures in a 1:1 ratio [20, 25–28]. Compared to panoramic radiographs, CBCT scans expose patients to higher levels of radiation. Multiple CBCT scans to continually monitor root position may not be recommended clinically, especially in children [27–29]. While CBCT technology has improved, resulting in decreased radiation dosage, practitioners are always recommended to follow the As Low as Reasonably Achievable (ALARA) principle and avoid exposing patients to radiation when possible [30]. Thus, a technique that can accurately monitor root position in three dimensions while also reduce radiation exposure to patients is desirable.

Recently, a new methodology, which generates an “expected root position” (ERP) setup, was demonstrated to have the potential to monitor root position at any stage of orthodontic treatment using a single pre-treatment CBCT scan [31, 32]. The generated ERP setup was reported to be an approximation of the root position at the orthodontic stage of interest, and it was demonstrated to be accurate in an ex vivo typodont model and clinically in one patient at post-treatment via color displacement maps. The ERP approach has not been demonstrated during orthodontic treatment where it has the potential to aid with bracket repositioning, especially at a designated reset appointment. The purpose of this study was to introduce the first application of generating an ERP setup during orthodontic treatment at the reset appointment.

## Materials and methods

**Fig. 1:**
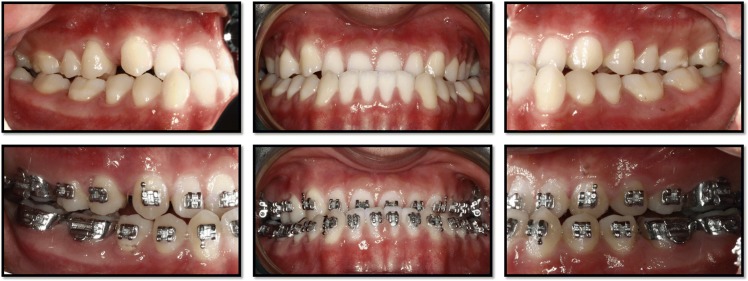
Clinical photographs of orthodontic treatment at pre-treatment (top) and at the reset appointment (bottom)

This retrospective study was approved by the Committee on Human Research of the University of California, San Francisco (UCSF). We obtained clinical records of a single patient who underwent treatment at the UCSF Division of Orthodontics and had casts and CBCT scans taken at pre-treatment and at the reset appointment. The patient was an 18-year-old Asian male with a skeletal and dental class III malocclusion treated with comprehensive orthodontic treatment and orthognathic surgery (Fig. 1). The reset appointment was performed prior to orthognathic surgery.

**Fig. 2:**
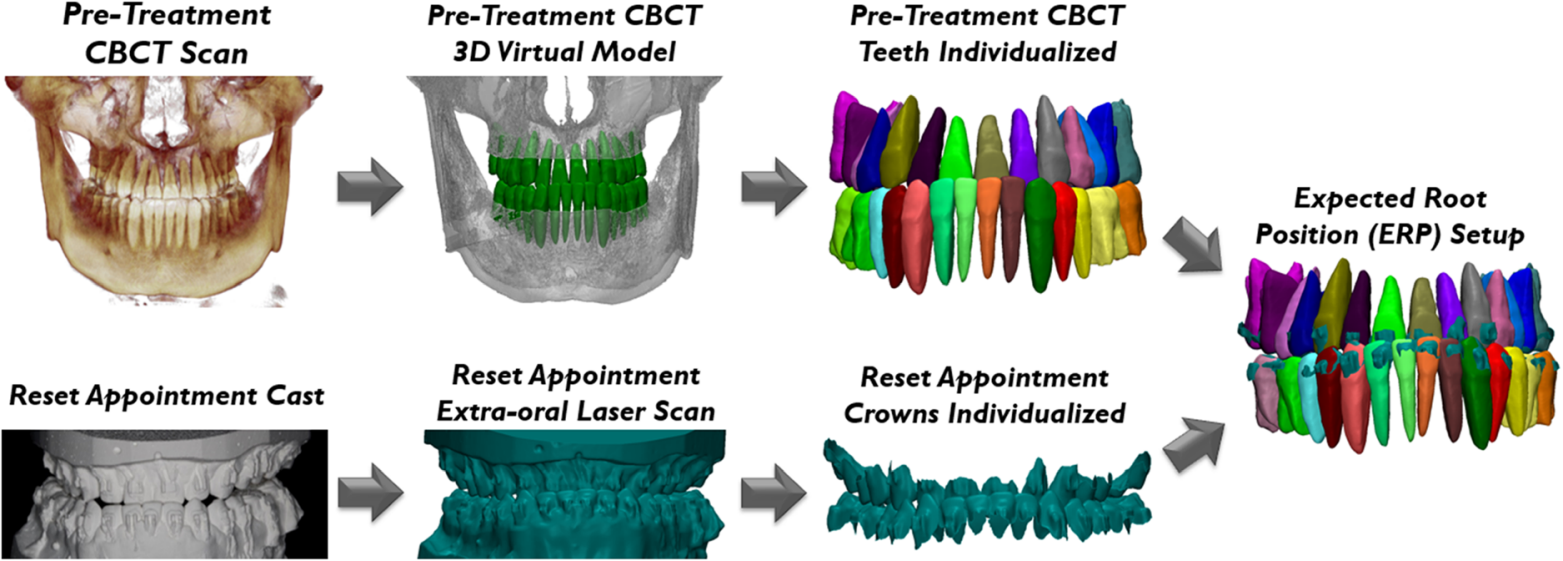
Generation of the ERP setup. The teeth from the pre-treatment CBCT scan are segmented and isolated. The reset appointment cast is scanned with an extra-oral laser scanner and individualized. The pre-treatment CBCT teeth are superimposed onto the reset appointment crowns yielding the ERP setup

Segmentations of teeth from CBCT scans taken at pre-treatment and at the reset appointment were performed using the Anatomodel modeling service (Anatomage, San Jose, CA). The cast taken at the reset appointment was scanned using an Ortho Insight (MotionView Software, LLC, Hixson, TN) extra-oral laser scanner. These laser-scanned crowns were segmented and exported as PLY files using the Ortho Insight software. The pre-treatment segmented CBCT teeth obtained from the Anatomodel modeling service were superimposed using 3-matic software (version 9.0; Materialise, Leuven, Belgium) onto their respective laser-scanned crowns yielding the ERP setup at the reset appointment (Fig. 2). This superimposition process first used the *N* points registration function to approximate the position of the crown of the pre-treatment CBCT tooth onto its respective laser-scanned crown by selecting three matching points on both crowns. Any gross errors in crown and root mesiodistal angulation and buccolingual inclination observed on the CBCT teeth after *N* points registration were corrected using the translation and rotation functions while roughly matching the alignment of the long axes of the CBCT teeth and laser-scanned crowns. The final part of this superimposition process utilized a global registration function which consisted of an iterative closest point algorithm. A color displacement map between the crowns of the pre-treatment CBCT teeth and the extra-oral laser scan of the reset appointment cast was generated to validate the accuracy of the superimposition.

**Fig. 3:**
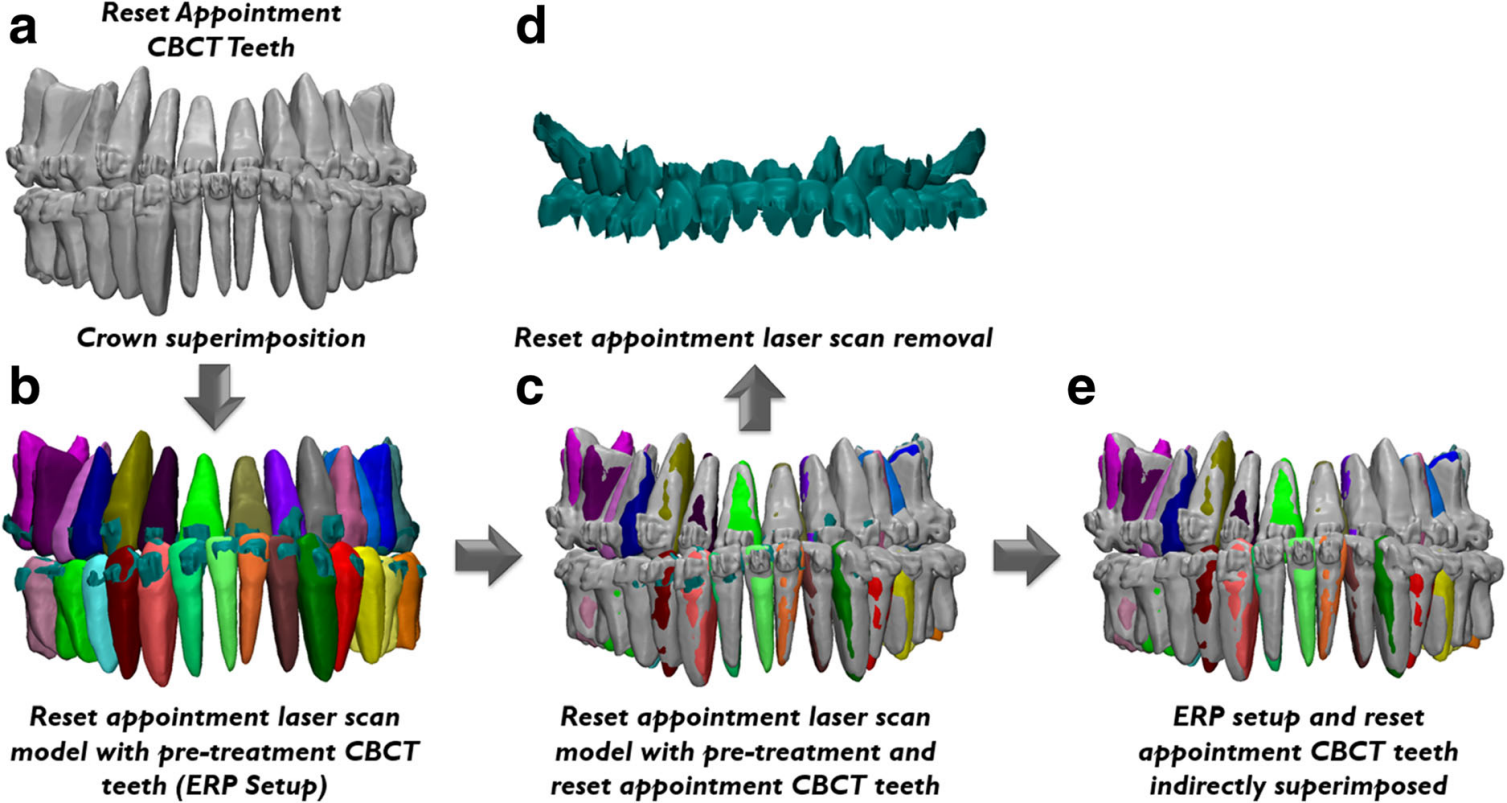
Indirect superimposition process: **a** reset appointment CBCT teeth; **b** reset appointment laser scan model with superimposed pre-treatment CBCT scan yielding the ERP setup; **c** reset appointment laser scan with both the superimposed pre-treatment and reset appointment CBCT teeth; **d** removal of the laser scan model from the viewport; **e** remaining ERP setup and reset appointment CBCT teeth indirectly superimposed allowing for comparison of the expected and true root positions

To validate the accuracy of the ERP setup, indirect superimposition was performed as described in literature [31, 32]. This was accomplished by superimposing the combined crowns of the CBCT teeth taken at the reset appointment (Fig. 3a) onto the crowns of the same extra-oral laser scan of the reset appointment cast used to generate the ERP setup (Fig. 3b). After this superimposition process, the crowns of the reset appointment CBCT teeth and the ERP setup were in the same position in three dimensions (Fig. 3c). After removing the reset appointment laser scan (Fig. 3d) from the three-dimensional viewport, the ERP setup and true position of the roots depicted by the reset appointment CBCT scan were now indirectly superimposed with each other (Fig. 3e). A color displacement map of the superimposed reset appointment CBCT teeth and extra-oral laser scan of the reset appointment cast was generated to validate the accuracy of the superimposition.

The ERP setup and reset appointment CBCT teeth were cut roughly at the cemento-enamel junction (CEJ) separating the roots and crowns. Color displacement maps were generated to study two scenarios: (1) superimposition of the ERP setup and reset appointment CBCT crowns and (2) superimposition of the ERP setup and reset appointment CBCT roots. All of the color displacement maps in this study were generated with the same parameters. Displacement within a 0.75-mm range was presented as green. Inward displacement greater than 0.75 mm of the laser-scanned crowns compared to the CBCT crowns is represented as blue and outward displacement greater than 0.75 mm as red.

## Results

**Fig. 4:**
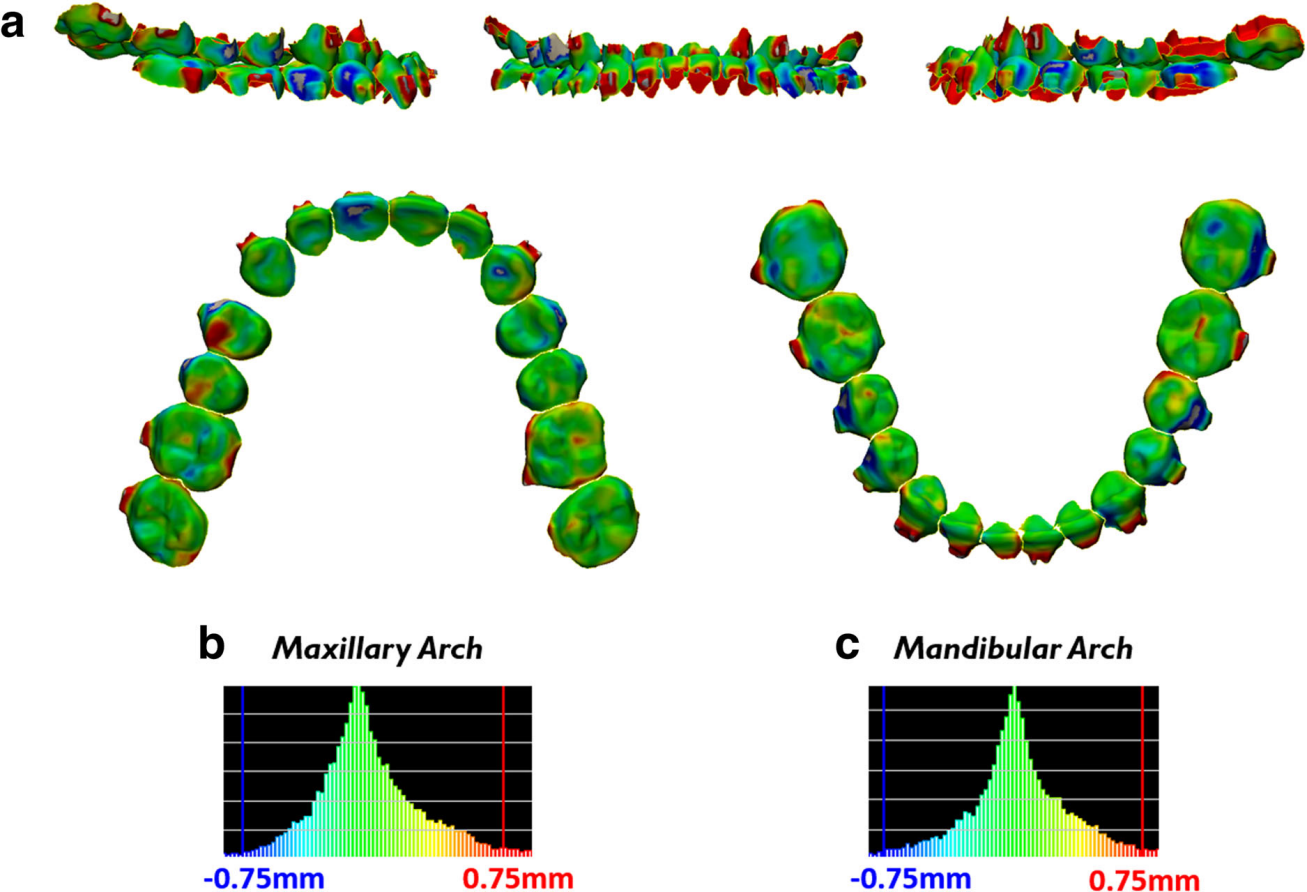
Verification of accurate crown superimposition during ERP setup generation after direct superimposition between the crowns of the pre-treatment CBCT teeth and the reset appointment laser scan. **a** Color displacement maps comparing the crown positions of the pre-treatment CBCT crowns and reset appointment laser scan crowns. Green areas indicate 0.0 mm displacement; blue and red areas indicate equal to or greater than 0.75 mm. **b**, **c** Histograms showing the distribution of displacements between crowns of the pre-treatment CBCT scan and reset appointment laser scan in the maxillary arch and mandibular arch

To verify accurate direct superimposition between the pre-treatment CBCT crowns onto the reset appointment laser scan crowns during generation of the ERP setup, a color displacement was generated. The color displacement map found that there was a maxillary displacement of 0.087 mm ± 0.328 mm with a maximum of 1.363 mm and mandibular displacement of 0.071 mm ± 0.382 mm with maximum of 1.398 mm (Fig. 4, Table 1).

**Table 1:** Color displacement map analysis

Analysis type	Mean displacement (mm)	Standard deviation (mm)	Maximum displacement (mm)
Pre-treatment CBCT crowns vs reset appointment laser scan crowns			
Maxillary crowns	0.087	0.328	1.363
Mandibular crowns	0.071	0.382	1.398
Reset appointment CBCT crowns vs reset appointment laser scan crowns			
Maxillary crowns	0.146	0.349	1.269
Mandibular crowns	0.289	0.508	1.999
ERP crowns vs reset appointment CBCT crowns			
Maxillary crowns	0.098	0.371	1.4
Mandibular crowns	0.203	0.438	1.848
ERP roots vs reset appointment CBCT roots			
Maxillary roots	0.021	0.396	1.429
Mandibular roots	0.079	0.499	1.786

**Fig. 5:**
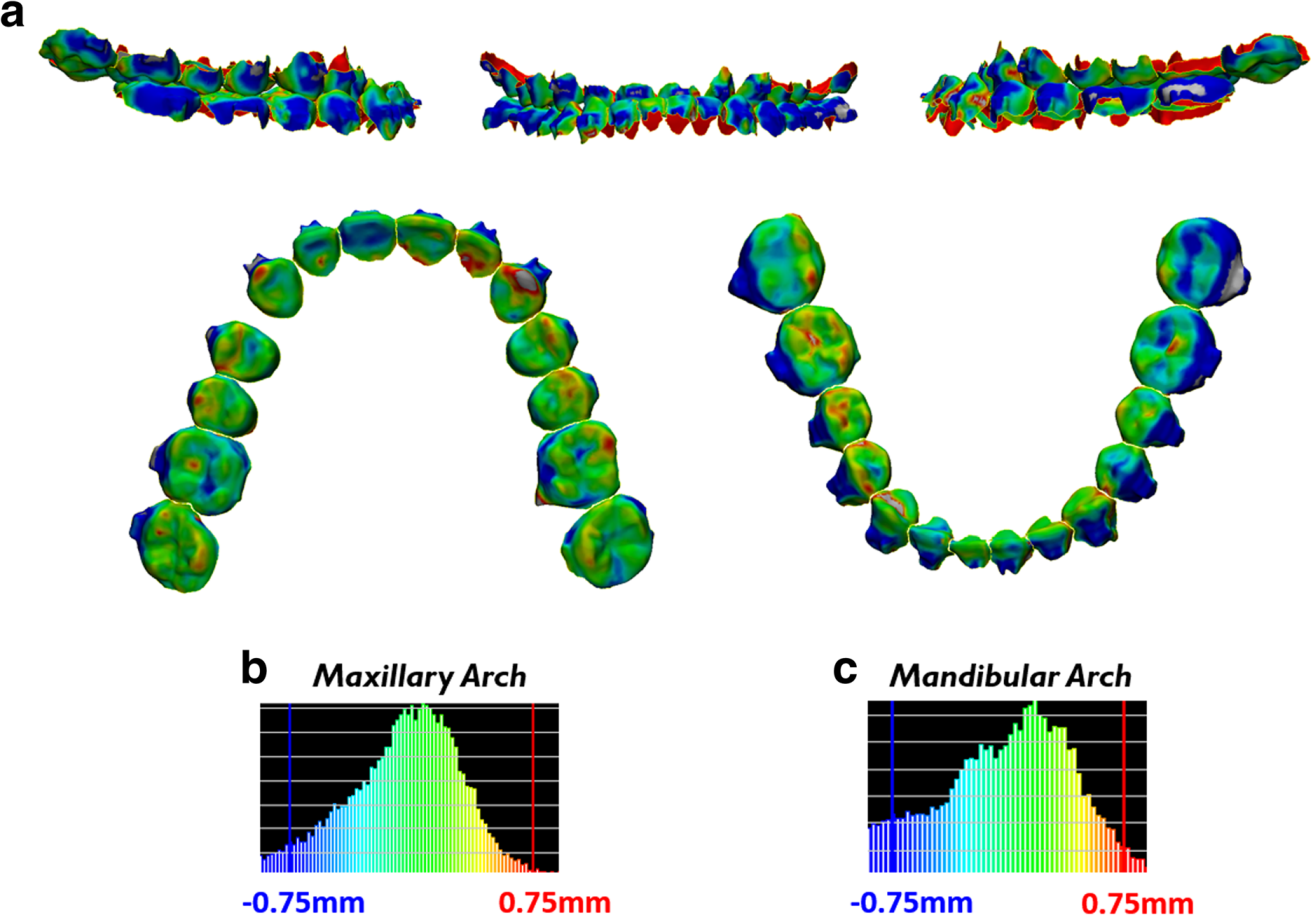
Verification of accurate crown superimposition after direct superimposition between the crowns of the reset appointment CBCT teeth and the reset appointment laser scan. **a** Color displacement maps comparing the crown positions of the reset appointment CBCT crowns and reset appointment laser scan crowns. Green areas indicate 0.0 mm displacement; blue and red areas indicate equal to or greater than 0.75 mm. **b**, **c** Histograms showing the distribution of displacements between crowns of the reset appointment CBCT scan and reset appointment laser scan in the maxillary arch and mandibular arch

Direct superimposition between the reset appointment CBCT crowns and reset appointment laser scan crowns was also verified to be accurate through a color displacement map. The color displacement map showed maxillary displacement of 0.146 mm ± 0.349 mm with a maximum of 1.269 mm and mandibular displacement of 0.289 mm ± 0.508 mm with a maximum of 1.999 mm (Fig. 5, Table 1).

**Fig. 6:**
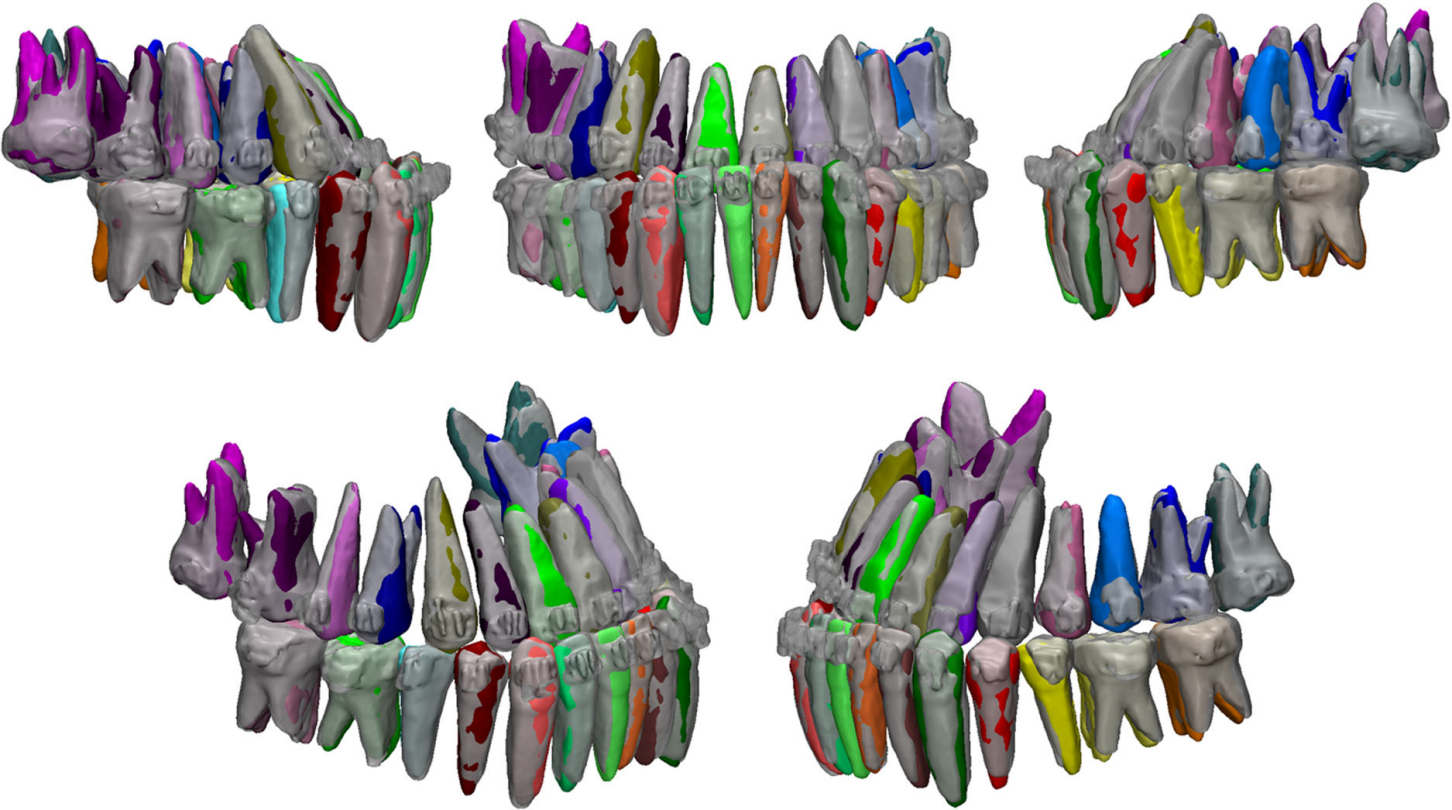
Qualitative comparison of the ERP setup (multicolored teeth) and the reset appointment CBCT teeth (transparent gray) after indirect superimposition

After indirect superimposition, the ERP setup was qualitatively compared to the reset appointment CBCT scan which served as the control. Figure 6 shows different viewpoints of the indirectly superimposed setups with the reset appointment CBCT virtual model semi-transparent. On a qualitative visual inspection, the root position generated by the ERP setup shows minimal differences compared to the true root positions depicted by the reset appointment CBCT roots.

**Fig. 7:**
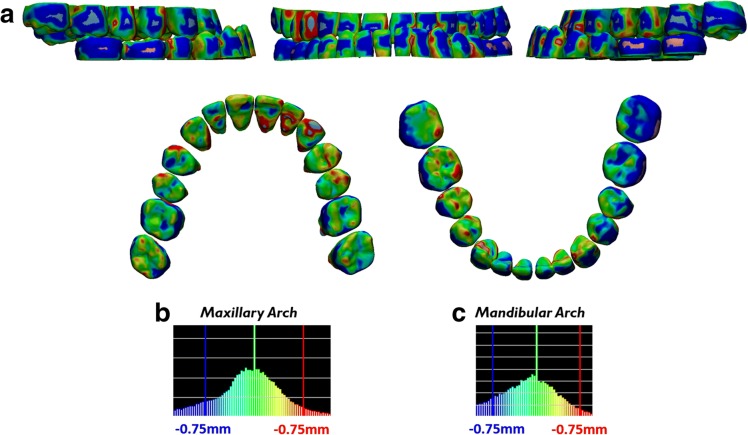
Verification of accurate crown superimposition after indirect superimposition of the ERP setup and reset appointment CBCT scan. **a** Color displacement maps comparing the crown positions of the ERP setup and reset appointment CBCT scan. Green areas indicate 0.0 mm displacement; blue and red areas indicate equal to or greater than 0.75 mm. **b**, **c** Histograms showing the distribution of displacements between crowns of the ERP setup and reset appointment CBCT scan in the maxillary arch and mandibular arch

**Fig. 8:**
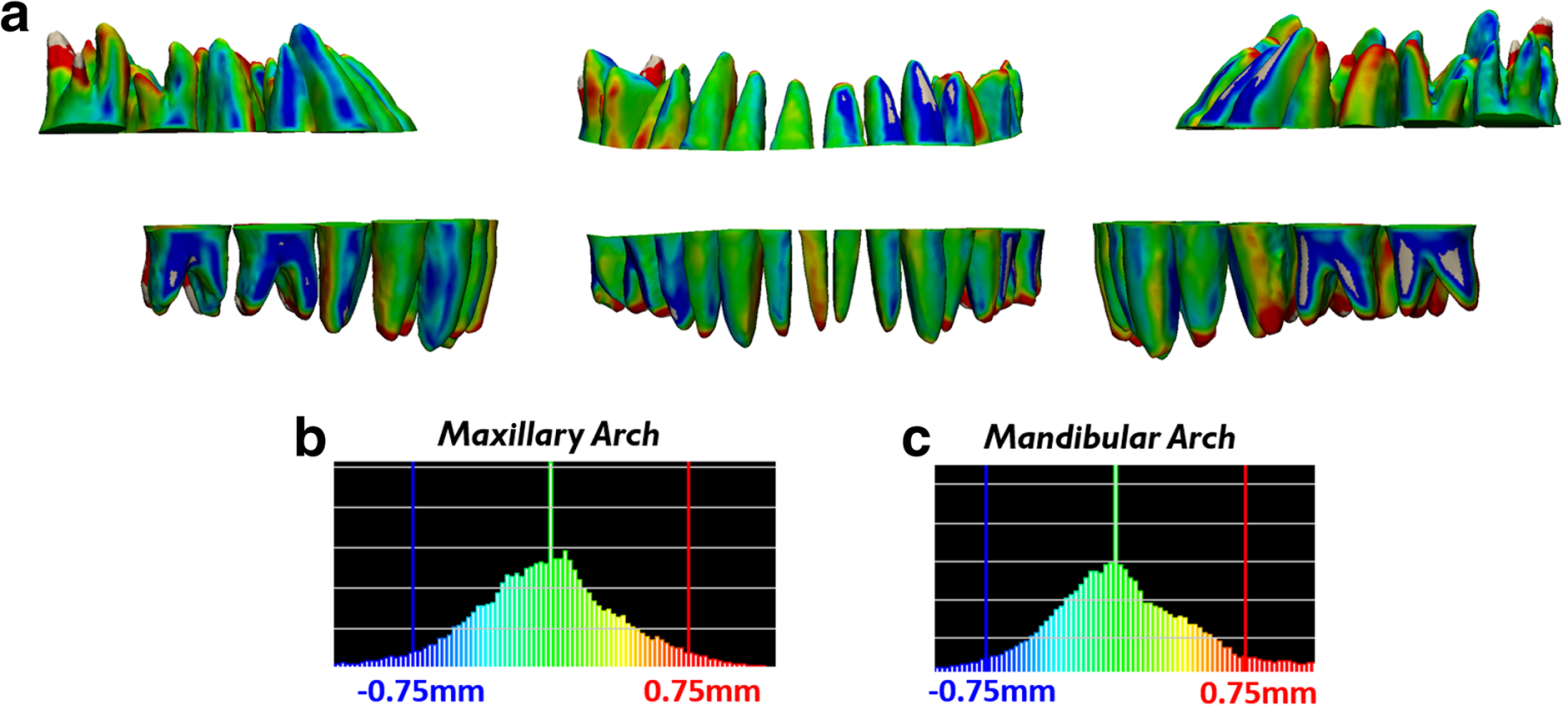
Measurement of displacements between the roots after indirect superimposition of the ERP setup and reset appointment CBCT scan. **a** Color displacement maps comparing the root positions of the ERP setup and reset appointment CBCT scan. Green areas indicate 0.0 mm displacement; blue and red areas indicate equal to or greater than 0.75 mm. **b**, **c** Histograms showing the distribution of displacements between roots of the ERP setup and reset appointment CBCT scan in the maxillary arch and mandibular arch

Color displacement map after indirect superimposition of the ERP setup crowns with the reset appointment CBCT crowns showed maxillary displacement of 0.098 mm ± 0.371 mm with a maximum of 1.400 mm and mandibular displacement of 0.203 mm ± 0.438 mm with a maximum of 1.848 mm (Fig. 7, Table 1). Color displacement map after indirect superimposition of the ERP setup roots with the reset appointment CBCT roots showed maxillary displacement of 0.021 mm ± 0.396 mm with a maximum of 1.429 mm and mandibular displacement of 0.079 mm ± 0.499 mm with a maximum of 1.786 mm (Fig. 8, Table 1).

## Discussion

Proper root position is necessary for successful orthodontic treatment that is stable, functional, and esthetic. Typically, the primary focus during orthodontic treatment is on crown position rather than root position because roots are not clinically visible and generally not directly involved with esthetics and occlusion [5, 7, 16]. Root position plays a role in periodontal health, restorative treatment, and occlusal function [2, 8–16]. Radiographs often reveal crown alignment errors in teeth with poor root angulation. Furthermore, the American Board of Orthodontics (ABO) recommends assessing root parallelism and deducts points if the roots of adjacent teeth are not parallel with each other or if they come in contact with each other [33]. The ABO recommends use of panoramic radiographs to monitor root alignment even though previous reports and the ABO have acknowledged that panoramic radiographs do not accurately depict root position [21–23]. Thus, a new approach that can accurately monitor root position would be desirable.

This study obtained digital models of the crowns via laser scans of poured up casts from the reset appointment. Previous reports have found that the accuracy of laser scans of poured up casts are comparable with intra-oral scans [34–38]. However, the accuracy of the digital model obtained from the extra-oral laser scan is dependent on an accurate impression and model pouring process. Therefore, to validate the accuracy of the extra-oral laser scan used in this study, direct superimposition comparing both the pre-treatment and reset appointment CBCT scan crowns to the reset appointment laser scan crowns was performed. Color displacement maps found minimal differences for both superimpositions.

To assess the accuracy of the root position depicted by the ERP setup, it was compared against the reset appointment CBCT scan which reflects the true root position. To minimize error in the analysis, an indirect superimposition process was performed in which the ERP setup and reset appointment CBCT scan were both superimposed onto the same reset appointment model laser scan. The indirect superimposition process is only applied for research purposes to use the reset appointment CBCT scan as a control. In a clinical setting, assessment of root position at the reset appointment using the ERP setup approach may eliminate the need for panoramic or CBCT imaging.

The accuracy of the indirect superimposition process was validated through color displacement map analysis of superimposed ERP setup and reset CBCT scan crowns in which minimal differences were found. The blue and red spots on the color maps were noted, indicating regions of displacement greater than 0.75 mm which appears primarily due to the presence of brackets and bands for the reset appointment CBCT scan. Sources of error in determining root form (size and shape) include noise, voxel size, contrast variance, and segmentation accuracy [39]. Occlusal anatomy is also often difficult to capture with threshold segmentation when the patient is in occlusion. A potential solution to this would be to have the patient bite into a thin piece of wax during the CBCT scan to create a small separation between the upper and lower teeth allowing for easier segmentation of the occlusal anatomy. Another potential solution would be to use a low-dose spiral CT scan, rather than a CBCT scan, since it has been shown to generate high-quality images for orthodontic diagnosis without a significant increase of radiation to patients [40]. However, even with the presence of brackets and bands, which also add noise during CBCT image acquisition, and some operator error during the threshold segmentation process, the ERP setup still was able to depict similar root position to the reset appointment CBCT scan.

This approach to generate an ERP setup was previously demonstrated in an ex vivo typodont model and at post-treatment [31, 32]. This study was the first to demonstrate this methodology during treatment to facilitate the correction of any root position errors. While radiographs at the reset appointment may still be needed to monitor root resorption and pathology, this study demonstrated that the ERP setup can be used, not just at the reset appointment, but at any time during treatment since the presence of bands and brackets does not appear to affect the accuracy of the ERP setup. This finding has clinical implications for practitioners who do not use a reset appointment in their treatment workflow because this demonstrates that they would be able to generate an ERP setup at any time during orthodontic treatment when they desire to evaluate root position. In addition, the ERP setup could potentially be generated at later appointments to monitor the root positions and to correct any root position errors that may not have been fully corrected in the reset appointment without any further radiation to the patient. Thus, this protocol may reduce the number of radiographic procedures recommended.

The main limitation of this methodology is that it is currently too time consuming for use in a clinical setting, though technology has improved the speed of this approach since the previous report of this method. Third-party vendors now exist that can perform the pre-treatment CBCT scan threshold segmentation for the practitioner which was previously the most time-consuming step. The superimposition process needed for each individual tooth is still a time-consuming step. However, intra-oral scan technology applies superimposition functions to stitch numerous snapshots of teeth together. Potentially in the future, intra-oral scanning technology may also be able to stitch the threshold segmentation of pre-treatment CBCT scan, obtained from the third party-vendor, in real time. Another limitation of this approach is that any change to the crown after the pre-treatment CBCT scan, such as a large restoration or crown, may make it difficult or impossible to perform the crown superimposition. If the crown superimposition cannot be performed, then the ERP setup for the tooth with the changed anatomy would not be possible to generate. Furthermore, teeth with restorations larger than two surfaces may also be difficult to segment out of the CBCT scan and could also potentially result in an inaccurate model of the tooth leading to unreliable crown superimposition.

## Conclusion

1. We have demonstrated the potential clinical use of the expected root position (ERP) approach to evaluate root position during orthodontic treatment without the need for additional radiation after a pre-treatment CBCT scan.
2. The bands and brackets during orthodontic treatment did not appear to affect the accuracy of the ERP setup.

## References

[1] Andrews LF (1972). The six keys to normal occlusion. Am J Orthod.

[2] Balut N, Klapper L, Sandrik J, Bowman D (1992). Variations in bracket placement in the preadjusted orthodontic appliance. Am J Orthod Dentofac Orthop.

[3] Miethke RR (1997). Third order tooth movements with straight wire appliances. Influence of vestibular tooth crown morphology in the vertical plane. J Orofac Orthop.

[4] Miethke RR, Melsen B (1999). Effect of variation in tooth morphology and bracket position on first and third order correction with preadjusted appliances. Am J Orthod Dentofac Orthop.

[5] Germane N, Bentley BE, Isaacson RJ (1989). Three biologic variables modifying faciolingual tooth angulation by straight-wire appliances. Am J Orthod Dentofac Orthop.

[6] Carlsson R, Rönnerman A (1973). Crown-root angles of upper central incisors. Am J Orthod.

[7] Bryant RM, Sadowsky PL, Hazelrig JB (1984). Variability in three morphologic features of the permanent maxillary central incisor. Am J Orthod.

[8] Vermylen K, De Quincey GNT, van’t Hof MA, Wolffe GN, Renggli HH (2005). Classification, reproducibility and prevalence of root proximity in periodontal patients. J Clin Periodontol.

[9] Vermylen K, De Quincey GNT, Wolffe GN, van’t Hof MA, Renggli HH (2005). Root proximity as a risk marker for periodontal disease: a case-control study. J Clin Periodontol.

[10] Olsen CT, Ammons WF, van Belle G (1985). A longitudinal study comparing apically repositioned flaps, with and without osseous surgery. Int J Periodontics Restorative Dent.

[11] Waerhaug J (1980). Eruption of teeth into crowded position, loss of attachment, and downgrowth of subgingival plaque. Am J Orthod.

[12] Nevins M (1982). Interproximal periodontal disease—the embrasure as an etiologic factor. Int J Periodontics Restorative Dent.

[13] Klassman B, Zucker HW (1969). Treatment of a periodontal defect resulting from improper tooth alignment and local factors. J Periodontol.

[14] Ritchey B, Orban B (1953). The crests of the interdental alveolar septa. J Periodontol.

[15] Akiyoshi M, Mori K (1967). Marginal periodontitis: a histological study of the incipient stage. J Periodontol.

[16] Dewel BF (1949). Clinical observations on the axial inclination of teeth. Am J Orthod.

[17] Sondhi A (2003). The implications of bracket selection and bracket placement on finishing details. Semin Orthod.

[18] Carlson SK, Johnson E (2001). Bracket positioning and resets: five steps to align crowns and roots consistently. Am J Orthod Dentofac Orthop.

[19] Keim RG, Gottlieb EL, Nelson AH, Vogels DS (2008). 2008 JCO study of orthodontic diagnosis and treatment procedures, part 1: results and trends. J Clin Orthod.

[20] Lagravère MO, Carey J, Toogood RW, Major PW (2008). Three-dimensional accuracy of measurements made with software on cone-beam computed tomography images. Am J Orthod Dentofac Orthop.

[21] Mckee IW, Glover KE, Williamson PC, Lam EW, Heo G, Major PW (2001). The effect of vertical and horizontal head positioning in panoramic radiography on mesiodistal tooth angulations. Angle Orthod.

[22] Garcia-Figueroa MA, Raboud DW, Lam EW, Heo G, Major PW (2008). Effect of buccolingual root angulation on the mesiodistal angulation shown on panoramic radiographs. Am J Orthod Dentofac Orthop.

[23] Owens AM, Johal A (2008). Near-end of treatment panoramic radiograph in the assessment of mesiodistal root angulation. Angle Orthod.

[24] Van Elslande D, Heo G, Flores-Mir C, Carey J, Major PW (2010). Accuracy of mesiodistal root angulation projected by cone-beam computed tomographic panoramic-like images. Am J Orthod Dentofac Orthop.

[25] Lascala CA, Panella J, Marques MM (2004). Analysis of the accuracy of linear measurements obtained by cone beam computed tomography (CBCT-NewTom). Dentomaxillofac Radiol.

[26] Hutchinson SY (2005). Cone beam computed tomography panoramic images vs. traditional panoramic radiographs. Am J Orthod Dentofac Orthop.

[27] Ludlow JB, Davies-Ludlow LE, Brooks SL, Howerton WB (2006). Dosimetry of 3 CBCT devices for oral and maxillofacial radiology: CB Mercuray, NewTom 3G and i-CAT. Dentomaxillofac Radiol.

[28] Brooks SL (2009). CBCT dosimetry: orthodontic considerations. Semin Orthod.

[29] Silva MAG, Wolf U, Heinicke F, Bumann A, Visser H, Hirsch E (2008). Cone-beam computed tomography for routine orthodontic treatment planning: a radiation dose evaluation. Am J Orthod Dentofac Orthop.

[30] Mah JK, Huang JC, Choo H (2010). Practical applications of cone-beam computed tomography in orthodontics. J Am Dent Assoc.

[31] Lee RJ, Pham J, Choy M, Weissheimer A, Dougherty HL, Sameshima GT (2014). Monitoring of typodont root movement via crown superimposition of single cone-beam computed tomography and consecutive intraoral scans. Am J Orthod Dentofac Orthop.

[32] Lee RJ, Weissheimer A, Pham J, Go L, de Menezes LM, Redmond WR (2015). Three-dimensional monitoring of root movement during orthodontic treatment. Am J Orthod Dentofac Orthop.

[33] Casko JS, Vaden JL, Kokich VG, Damone J, James RD, Cangialosi TJ (1998). Objective grading system for dental casts and panoramic radiographs. American Board of Orthodontics. Am J Orthod Dentofacial Orthop.

[34] Seelbach P, Brueckel C, Wöstmann B (2013). Accuracy of digital and conventional impression techniques and workflow. Clin Oral Investig.

[35] Flügge TV, Schlager S, Nelson K, Nahles S, Metzger MC (2013). Precision of intraoral digital dental impressions with iTero and extraoral digitization with the iTero and a model scanner. Am J Orthod Dentofac Orthop.

[36] Ender A, Mehl A (2013). Influence of scanning strategies on the accuracy of digital intraoral scanning systems. Int J Comput Dent.

[37] Ender A, Mehl A (2011). Full arch scans: conventional versus digital impressions—an in-vitro study. Int J Comput Dent.

[38] Naidu D, Freer TJ (2013). Validity, reliability, and reproducibility of the iOC intraoral scanner: a comparison of tooth widths and Bolton ratios. Am J Orthod Dentofac Orthop.

[39] Hatcher DC (2010). Operational principles for cone-beam computed tomography. J Am Dent Assoc.

[40] Cordasco G, Portelli M, Militi A, Nucera R, Lo Giudice A, Gatto E, Lucchese A (2013). Low-dose protocol. Prog Orthod.

